# Supplementary material for: Hypoglycemia prevention practice and its associated factors among diabetes patients at university teaching hospital in Ethiopia: Cross-sectional study
Source: PLoS One. 2020 Aug 21;15(8):e0238094. doi: 10.1371/journal.pone.0238094 (PMC7446928; doi:10.1371/journal.pone.0238094)
Supplement: S1 File — (DOCX) [file pone.0238094.s001.docx]

| **Socio demographic and clinical characteristics** | | |
| --- | --- | --- |
| **Question** | | **Response** |
| 1 | Age in years | --------------------------------- |
| 2 | Gender | A. Male B. Female |
| 3 | Religion? | A. Orthodox B. Islam  C. protestant D. catholic  E. Other |
| 4 | Residence? | A. Rural B. Urban |
| 5 | Marital status | A. Unmarried B. Married  C. Separated D. Divorce  E. Widowed |
| 6 | Educational status | a. Primary education  b. Secondary education  c. Graduate  d. unable to write and read |
| 7 | Occupation | a. Unemployed  b. Private employed  c. Government employed  d. student  e. retired |
| 8 | Income per month | a. Below 500 birr  b. 500 – 1000 birr  c. 1001 – 1500 birr  d. Above 1500 birr |
| 9 | Any substance use | ………………………….. |
| 10 | What is your current BMI | ……………………….. |
| 11 | What type of diabetic are you? | a. type I b. type II |
| 12 | Diabetes duration in years | ------------------------------- |
| 13 | Types of treatment (pharmacologic) | ----------------------------- |
| 14 | Frequency of taking medication | 1. Once a day 2. Twice a day 3. Thrice a day |
| 15 | Do you have any history of hypoglycemia with in last month? | a. Yes  b. No |
| 16 | What is your current blood glucose level? | ------------------------------- |
| 17 | Any comorbid condition |  |
| **Part two: knowledge on prevention of hypoglycemia** | | |
| 1 | What is hypoglycemia?  Blood sugar level is ________ | 1. Less than 70 mg/dl 2. 70- 110 mg/dl 3. 110-150mg/dl 4. More than 150 mg/dl |
| 2 | What is the normal fasting blood sugar level?  Blood sugar level is _________ | 1. Less than 70 mg/dl 2. 70-110 mg/dl ‘ 3. 120-150 mg/dl 4. 150 -300 mg/dl |
| 3 | What are the main causes of hypoglycemia? | 1. Irregular meal time 2. Unplanned exercise 3. Excessive alcohol consumption 4. Intake of carbohydrate food |
| 4 | What is the risk factor for hypoglycemia in diabetes? | a. Usage of high dosage of anti-diabetic drugs  b. Usage of low dose of insulin  c. Intake of more fluids  d. Intake of more sugar content foods |
| 5 | What are the early symptoms of hypoglycemia? | a. Frequent thirst, frequent urination and frequent intake of water  b. Excessive perspiration, palpitations and body shaking  c. Body pain, breathlessness and stomach pain  d. Decreased pulse rate, giddiness and vomiting |
| 6 | What is the symptom of night time hypoglycemia? | a. Body pain  b. Mood irritability  c. Sleeplessness  d. Clammy neck |
| 7 | What is the complication of hypoglycemia? | a. Loss of consciousness  b. Dehydration  c. Breathlessness  d. Gastrointestinal bleeding |
| 8 | What is the way to prevent hypoglycemia? | a. Avoid starving more than 4-5 hours  b. Avoiding snacks in-between meals  c. Avoiding snacks in-between exercises  d. Avoiding drinking water |
| 9 | What is the way of prevent night time hypoglycemia? | a. Taking extra glucose containing snacks before going to bed  b. Starve before going to bed  c. Drinking more amount of water  d. Drinking milk before bed time |
| 10 | Which is the best way to prevent repeated hypoglycemia? | a. The person should starve for the day  b. The person should eat more sugar containing food  c. The person should have the usual meal that is due at the time  d. The person should exercise immediately |
| **Part three: Practice regarding hypoglycemia prevention** | | |
| 1 | What precaution you should take to avoid hypoglycemia while travelling? | a. Carrying sugar or sugar candy  b. Carrying a glass of milk  c. Carrying water  d. Carrying biscuits  e. Carrying soft drinks like mirinda |
| 2 | Which of the following is the self - management for hypoglycemia? | a. Taking 15 gms of sugar  b. Fruits like papaya in between the meals  c. Taking 500 ml of water  d. Taking cheese and butter |
| 3 | When did you experience hypoglycemic episode? | a. skipping meals b. During exercise  c. Early morning d. Night time |
| 5 | How long diabetic patients should do exercise? | a. Up to 15 minutes b. 30 minutes to 1 hour  c. 1hour to 1.30hour d. Up to 2 hours |
| 6 | Which type of exercise is safe and beneficial for diabetic patients to avoid Hypoglycemia? | a. Walking b. Weight lifting  c. Running d. Jogging |
| 7 | What is the effect of weight lifting exercise in hypoglycemic patients? | a. Maintains the blood sugar level  b. Lowers the blood sugar level  c. Increases the blood cholesterol level  d. Increases the blood sugar level |
| 8 | Did you self-monitor your blood glucose at home? | 1. Yes b. no |
| 9 | Did you measure your  glucose levels when you think you are hypoglycemic? | a. yes b. no |
| 10 | When should we retest blood sugar level after the treatment of hypoglycemia? | a. After 5 minutes  b. After 10 minutes  c. After 20 minutes  d. After 15 minutes |
| 11 | Did you take snack between meals? | 1. Yes b. no |
| 12 | Did you Take carbohydrate diets irregularly | 1. Yes b. no |
| 13 | Coming in regular appointments | a. Yes b. no |
| 14 | Did you Adjustment your medication based on your symptoms and blood glucose level? | 1. Yes b. no |
| 15 | Did you Report low sugar episode to the doctor | a. yes b. no |

Annex 2: questionnaire Amharic version

**የፍቃደኝነት ማረጋገጫ ቅፅ**

ዩኒቨርሲቲ ፣ ጤና ሳይንስ ኮሌጅ

መጠይቁን ሙሉ ለሙሉ ያለመሙላት ወይንም የማቋረጥ መብትዎም የተጠበቀ ነዉ፡፡

በጥናቱ ዉስጥ ለመሳተፍ ፈቃደኛ ነዎት?

1. አዎ ፊርማ _____________ ቀን ________

የመጠይቁ ሰብሳቢ ፊርማ_________ቀን _______

2. አይደለሁም ---- እባክዎን እዚህ ጋር ይቁሙ

በጥናቱ ዉስጥ ለመሳተፍ ፈቃደኛ ከሆኑ ትክክለኛ የሆነ መልስ እንዲሰጡን በትህትና እንጠይቃለን፡፡

**ስለትብብርዎ በቅድሚያ እናመሰግናለን!!!**

**የደም የስካር መጠን ማነስን መከላከል የተመለከተ ጥናት**

የታካሚዉ መለያ ቁጥር-----------------------------------------

| **ክፍል አንድ፡ አጠቃላይ ግላዊ መረጃዎችን የሚመለከቱ ጥያቄዎች** | | | | | |
| --- | --- | --- | --- | --- | --- |
| **ጥያቄ** | | | **ምላሽ** | | |
| 101 | | ፆታ? | ሀ.ወንድ ለ. ሴት | | |
| 102 | | እድሜዎት ስንት ነዉ? | -------------አመት | | |
| 103 | | ሐይማኖትዎ ምንድን ነዉ? | ሀ. ኦርቶዶክስ ለ. ሙስሊም  ሐ. ፕሮቴስታንት መ. ካቶሊክ  ሠ. ሌላ ካለ | | |
| 104 | | የሚኖሩበት ቦታ? | ሀ. ከተማ ለ. ገጠር | | |
| 105 | | የጋብቻ ሁኔታዎ እንዴት ነዉ? | ሀ. ያላገባ ለ. ያገባ ሐ. የፈታ መ. በሞት ምክንያት የተልያዩ | | |
| 106 | | የትምህርት ደረጃ? | ሀ. የመጀመሪያ ደረጃ ለ. ሁለተኛ ደረጃ  ሐ. የተመረቀ መ. መጻፍና ማንበብ የማይችል | | |
| 107 | | የስራ ሁኔታ? | ሀ. ስራ የሌለው ለ. የግል ሰራተኛ  ሐ. የመንግስት ሰራተኛ መ. ተማሪ  ሠ. ሌላ ካለ | | |
| 108 | | የወር የገቢ መጠን ? | ሀ. ከ500 ብር በታች ለ. ከ500-1000 ብር  ሐ. ከ1001-1500 ብር መ. ከ1500ብር በላይ | | |
| 109 | | የደባል ሱስ አለ (አዎ ካሉ ምን) ? | .......................... | | |
| 110 | | አሁን BMIዎ ስንት ነው ? | ......................... | | |
| 111 | | የየትኛው የስካር ህመም አይነት ተጠቂ ነዎት ? | ሀ. ታይፕ 1 ለ. ታይፕ 2 | | |
| 112 | | የስካር ህመም እንዳለብዎ ካወቁ ምን ያህል ዓመት ሆነዎት? | ...................ዓመት | | |
| 113 | | የሚወስዱት ህክምና (መድሃኒት) ዓይነት ምንድን ነው ? | ................... | | |
| 114 | | መድሃኒትዎን በቀን ስንት ጊዜ ይወስዳሉ ? | ሀ. አንድ ጊዜ ለ. ሁለት ጊዜ  ሐ. ሶስት ጊዜ | | |
| 115 | | ባለፈው ወር ውስጥ የደም የስካር መጠን ማነስ አጋጥወታል ? | ሀ. አዎ  ለ. አይ | | |
| 116 | | አሁን ላይ ያለዎት የደም የስካር መጠን ስንት ነው ? | .................. | | |
| 117 | | ተጨማሪ ህመም አይነት አለብዎ (ካለ ምን) ? | ................ | | |
| **ክፍል ሁለት፡ ስለደም የስካር መጠን ማነስ መከላከል ያለ እውቀት መገምገሚ** | | | | | |
| 201 | የደም የስካር መጠን ማነስ ማለት ምን ማለት ነው ?  የደም የስካር መጠን ................... ሲሆን | | | ሀ. ከ70 ሚሊግራም/ዴሲሊትር በታች  ለ. ከ70-110 ሚሊግራም/ዴሲሊትር  ሐ. ከ110-150 ሚሊግራም/ዴሲሊትር  መ. ከ150ሚሊግራም/ዴሲሊትር በላይ | |
| 202 | ጤናማ የሆነው ከቁርስ በፊት ሚወሰድ የደም የስካር መጠን ስንት ነው ?  የደም የስካር መጠን ................... ሲሆን | | | ሀ. ከ70 ሚሊግራም/ዴሲሊትር በታች  ለ. ከ70-110 ሚሊግራም/ዴሲሊትር  ሐ. ከ120-150 ሚሊግራም/ዴሲሊትር  መ. ከ150-300ሚሊግራም/ዴሲሊትር | |
| 203 | ዋና የደም የስካር መጠን ማነስን ምክኒያት የሆነው የትኛው ነው ? | | | ሀ. ወጥ ያልሆነ የመመገቢያ ሰዓት  ለ. ያልታሰብ የሰውነት እንቅስቃሴ  ሐ. መጠኑ የበዛ አልኮል መውሰድ  መ. ኃይል ሰጪ የሆነ ምግብ መውሰድ | |
| 204 | ለደም የስካር መጠን ማነስ አጋላጭ የሆኑ ሁኔታዎች ምንድን ናቸው ? | | | ሀ. የስካር መድሃኒቶችን አብዝቶ መውሰድ  ለ. ትንሽ የእንሱሊን መጠን መውሰድ  ሐ. ብዙ ፈሳሽ መውሰድ  መ. ስካርነት ያለው ምግብ በብዛት መውሰድ | |
| 205 | ቅድሚያ የሚታዩ የደም የስካር መጠን ማነስ ምልክቶች ምንድን ናቸው ? | | | ሀ. ቶሎ ቶሉ ውሃ መጠማት፣ቶሎ ቶሎ መሽናት እና ቶሎ ቶሎ ውሃ መጠጣት  ለ. በብዛት ማላብ፣ የልብ ምት መሰማት እና የሰውነት መንቀጥቀጥ  ሐ. የሰውነት ህመም፣ የትንፋሽ ማጠር እና የሆድ ህመም  መ. የልብ ምት መቀነስ፣ ብዥታ እና ማስታወክ | |
| 206 | በሌሊት የሚከሰት የደም የስካር መጠን ማነስ ምልክቶች ምንድን ናቸው ? | | | ሀ. የሰውነት ህመም  ለ. የጸባይ መቀያየር  ሐ. የእንቅልፍ ማጣት  መ. አንገት | |
| 207 | የተባባሰ የደም የስካር መጠን ማነስ ምን ሊያስከትል ይችላል ? | | | ሀ. እራስን መሳት  ለ. የሰውነት ፈሳሽ ማነስ  ሐ. የትንፋሽ ማጠር  መ. የጨጉራ መድማት | |
| 208 | የደም የስካር መጠን ማነስን እንዴት መከላከል ይቻላል ? | | | ሀ. ምግብ ሳይወስዱ ከ4-5 ሰዓት በላይ አለመቆየት  ለ. በዋና ምግቦች መካከል ተጨማሪ ምግብ አለመውሰድ  ሐ. በሰውነት እንቅስቃሴዎች መካከል ተጨማ ምግቦችን አለመወሰድ  መ. ውሃ አለመጠጣት | |
| 209 | በሌሊት የሚከሰት የደም የስካር መጠን ማነስ እንዴት መከላከል ይቻላል ? | | | ሀ. ወደ መኝታ ከመሄድ በፊት ተጨማሪ ስካርነት ያላቸው ምግቦችን መውሰድ  ለ. ወደ መኝታ ከመሄድ በፊት ምግብ አለመውሰድ  ሐ. ተጨማሪ ውሃ መጠጣት  መ. ወደ መኝታ ከመሄድ በፊት ወተት መጠጣት | |
| 210 | ተደጋጋሚ የሆነ የደም የስካር መጠን ማነስ እንዴት መከላከል ይቻላል ? | | | ሀ. ቀኑን ሙሉ ምግብ አለመውሰድ  ለ. ብዙ ስካርነት ያላቸውን ምግቦች መመገብ  ሐ. የተለመደ የምግብ አይነቶችን በተለመደው ሰዓት መውሰድ  መ. ወዲያውኑ የሰውነት እንቅስቃሴ ማድረግ | |
| **ክፍል ሶስት፡ የደም የስካር መጠን ማነስን ለመከላከል የሚደረጉ ተግባራት መገምገሚያ** | | | | | |
| 301 | በጉዞ ወቅት የደም የስካር መጠን ማነስን ለመከላከል ምን ቅድሚያ ጥንቃቄ ያደርጋሉ ? | | | | ሀ. ስካር ወይም ከረሜላ መያዝ  ለ. ወተት መያዝ  ሐ. ውሃ መያዝ  መ. ብስኩት መያዝ  ሠ. እንደ ሚሪንዳ ያሉ ለስላሳ መጠጦችን መያዝ |
| 302 | የደም የስካር መጠን ማነስን እንዴት በራስዎ ማከም ይችላሉ ? | | | | ሀ. 15 ግራም የሚሆን ስካር መውሰድ  ለ. በዋና ምግቦች መካከል እንደ ፓፓያ ያሉ ተጨማሪ ምግቦችን መውሰድ  ሐ. ግማሽ ሊትር ውሃ መጠጣት  መ. ቅቤ እና አይብ መውሰድ |
| 303 | መቸ የደም የስካር መጠን ማነስ ያጋጥመወታል ? | | | | ሀ. ምግብ ሳይወስዱ ሲቀሩ  ለ. የሰውነት እንቅስቃሴ በሚያደረጉበት ወቅት  ሐ. ጥዋት ላይ  መ. ሌሊት ላይ |
| 304 | የስካር ህመምተኞት ለምን ያህል ጊዜ የሰውነት እንቅስቃሴ ማድረግ አለባቸው ? | | | | ሀ. እስከ 15 ደቂቃ  ለ. ከ30 ደቂቃ እስከ 1 ሰዓት  ሐ. ከ1 ሰዓት እሰከ 1 ሰዓት ተኩል  መ. እስከ 2 ሰዓት |
| 305 | የደም የስካር መጠን ማነስን ለመከላከል የትኛው የሰውነት እንቅስቃሴ ለስካር ህመምተኞት ጠቃሚ እና የማይጎዳ ነው ? | | | | ሀ.በእግር መራመድ  ለ.ክብደት ማንሳት  ሐ. መሮጥ  መ. የሶምሶማ ሩጫ |
| 306 | ክብደት ማንሳትን ያካተተ የሰውነት እንቅስቃሴ የደም የስካር መጠናቸው አነስተኛ ለሆኑ ህመምተኞች ምን ሊያስከትል ይችላል ? | | | | ሀ. በደም የስካር መጠናቸውን አይለውጥም  ለ. የደም የስካር መጠንን ይቀንሳል  ሐ. የደም የስብ መጠንን ይጨምራል  መ. የደም የስካር መጠንን ይጨምራል |
| 307 | በቤትዎ ውስጥ የደም የስካር መጠንዎን ይለካሉ ? | | | | ሀ. አዎ  ለ. አይ |
| 308 | የደም የስካር መጠንዎ ያነሰ ሲመስልዎት የደም የስካር መጠንውን ይለካሉ ? | | | | ሀ. አዎ  ለ. አይ |
| 309 | የደም የስካር መጠን ማነስ ካጋጠምዎ እና ከታከሙ በኃላ መቸ በድጋሚ የስካር መጠንዎን መለካት አለብዎት ? | | | | ሀ. ከ5 ደቂቃ በኃላ ለ. ከ10 ደቂቃ በኃላ  ሐ. ከ15 ደቂቃ በኃላ መ.ከ20 ደቂቃ በኃላ |
| 310 | በዋና ምግቦች መካከል ተጨማሪ ምግብ ይወስዳሉ ? | | | | ሀ. አዎ  ለ. አይ |
| 311 | ወጥ ያልሆነ የሀይል ሰጪ ምግቦች አመጋገብ ይከተላሉ ? | | | | ሀ. አዎ  ለ. አይ |
| 312 | በህክምና ቀጠርዎ ሰዓት ይመጣሉ? | | | | ሀ. አዎ  ለ. አይ |
| 313 | የሚወስዱትን የመድሃኒት መጠን በደም የስካር መጠንዎና ምልክቶች አማካኝነት ያስተካክላሉ? | | | | ሀ. አዎ  ለ. አይ |
| 314 | የደም የስካር መጠን ማነስ ሲያጋጥምዎ ለሃኪምዎት ያስታውቃሉ? | | | | ሀ. አዎ  ለ. አይ |
