# Supplementary material for: Hypoglycemia prevention practice and its associated factors among diabetes patients at university teaching hospital in Ethiopia: Cross-sectional study
Source: PLoS One. 2020 Aug 21;15(8):e0238094. doi: 10.1371/journal.pone.0238094 (PMC7446928; doi:10.1371/journal.pone.0238094)
Supplement: Attachment — Submitted filename: Rebuttal letter.docx [file pone.0238094.s002.docx]

**Response to reviewers**

Reviewer 1

- The introduction presents a nice review on hypoglycemia, but it needs to better connect to relevant literature in the area of knowledge and practice of preventing hypoglycemia.
- I revised the introduction part of the manuscript to include literatures about knowledge and practice of hypoglycemia prevention
- The justification for conducting this study is lacking.

Despite the abundance of studies on self-care practice and knowledge about hypoglycemia, there are no recent studies done on knowledge and practice regarding hypoglycemia prevention among diabetic patients especially in the study area. So this study tried to identify the current level of both knowledge and practice of diabetic patient’s towards prevention of hypoglycaemia and based on the finding we will recommend appropriate measure to be taken to have a better prevention of hypoglycaemia.

- In the final paragraph the authors refer to previous studies – this should be expanded and these studies should be referenced.
- We revised it to elaborate the idea and also cited.
- The methods – in study area and period “Diabetic patients get service two days per week” is unclear
- In our hospital the ambulatory care setting is not so mach sefficent due to spece so the management of the hospital try to adress these issue by distributing the most common cases in the working days. So diabetic patients get out patient or ambulatory care service two days per week (Tuesday and Friday). By the way it is temphorary solution in the near future there will be separate DM clinic that serves full time for DM patients only.
- About data collection tool—>it ‘s validation cutoff point to say good or pooris not clear and not referenced what is the mean score elaborate it
- I revised based on the comment to resolve the ambiguity. It was adapted from previous studies and also validated I put the reference for these issues.
  - Knowledge on hypoglycemia prevention was assessed using 10 questions with a maximum of 12 points score which was calculated by given one for each correct response and zero for each wrong response. Hypoglycemia prevention practice was determined using 15 questions with a maximum of 17 correct responses which was calculated by giving one for each correct response and zero for each wrong response. So the cut point to say good or poor is 50% of the maximum score if someone score more than 50% of the total score of knowledge as well as practice questions he had good knowledge or practice.
- Results – data in the figures is also presented in the table and text. Results should be selectively presented in a single format.
- I revised table and remove the figure based on your recommendation.
- The discussion does not connect the present study to previous findings. The discussion is insufficient due to lack of connection to justification.
- I worked it try to connect justify the possible reason for the discrepancies. Still due to lack of study in our country there may not sufficient literatures published.

Reviewer 2

- The relationship between a patient’s knowledge score and their practice score could be investigated with these data (but is not).
- I worked the analysis to see the association factor for hypoglycemia prevention and I got knowledge was the most important significant predictor of practice of hypoglycemia prevention.
- About data collection tool—>it‘s validation cutoff point to say good or poor is not clear what is the mean score elaborate it. Tables and figure needs revision.
- I revised based on the comment to resolve the ambiguity. It was adapted from previous studies.

Good Knowledge: when patients respond the half or above the half score of the maximum correct response (score ≥ 6) of the knowledge questions. And the reverse is true for poor

Good Practice: when patients respond the half or above the half score of the maximum correct response (score ≥8.5) of the practice questions.

Tables were revised to address your comment by the way it was type error there was there. Figures were deleted.

- About discussion it was unintelligible and so many topographical and type error so it needs revision -🡪 I did it again to be clear and understood.
- Multiple references are not cited in the manuscript🡪 I apologize totally I revised it.
