# Supplementary material for: Hypoglycemia prevention practice and its associated factors among diabetes patients at university teaching hospital in Ethiopia: Cross-sectional study
Source: PLoS One. 2020 Aug 21;15(8):e0238094. doi: 10.1371/journal.pone.0238094 (PMC7446928; doi:10.1371/journal.pone.0238094)
Supplement: Attachment — Submitted filename: Response to reviewer.docx [file pone.0238094.s003.docx]

Response to reviewer

1. Introduction should include predictors to hypoglycemia prevention practice.

- As far as investigators knowledge there is no literatures which showed predictors of hypoglycemia prevention practice except reference 21 and 26. And these reference are reviewed under paragraph 8 under introduction.

2. Operational definition still unclear?

Now it becomes clarified

- Good Knowledge: when patients scored > 6 on the knowledge assessment questions.
- Poor Knowledge: when patients scored < 6 on the knowledge assessment questions.
- Good Practice: when patients scored >8.5 on the practice assessment questions.
- Poor practice: when patients scored < 8.5 on the practice assessment questions.

3. Economic status should be stratified based on the average income and change to international currency.

- This stratification is based on the study area salary scale. But we changed with USD by considering current currency exchange.

4. These findings are limited to the region under study, but it's also limited to patients that regularly visit the hospital.

- Accepted and stated to limitation part this study.
